# Selection of potential reference genes for RT-qPCR in the plant pathogenic fungus *Colletotrichum fructicola*

**DOI:** 10.3389/fmicb.2022.982748

**Published:** 2022-08-08

**Authors:** Xingzhou Chen, Xinggang Chen, Qian Tan, Yuan He, Zhikai Wang, Guoying Zhou, Junang Liu

**Affiliations:** ^1^Key Laboratory of Cultivation and Protection for Non-Wood Forest Trees, Ministry of Education, Central South University of Forestry and Technology, Changsha, China; ^2^Key Laboratory of National Forestry and Grassland Administration on Control of Artificial Forest Diseases and Pests in South China, Central South University of Forestry and Technology, Changsha, China; ^3^Hunan Provincial Key Laboratory for Control of Forest Diseases and Pests, Central South University of Forestry and Technology, Changsha, China

**Keywords:** *Colletotrichum fructicola*, RT-qPCR, *Camellia oleifera*, reference gene, transcriptome

## Abstract

*Colletotrichum* is widespread, and these pathogenic fungi can cause various plant diseases. Studies have shown that *Colletotrichum fructicola* cause oil-tea (*Camellia oleifera*) anthracnose and is widely distributed as a dominant fungus in all *Ca. oleifera*-producing regions. Real-time quantitative PCR(RT-qPCR) is considered the most reliable technique for simultaneously measuring relative gene expression levels in different tissues. Target genes are typically quantified using RT-qPCR to explore gene function, and reliable RT-qPCR results require data normalization using stable reference genes. No studies have reported a suitable reference gene in *C. fructicola*. This study has eight candidate reference genes (*CfCk*, *CfRpp*, *CfUce*, *CfRrp*, *CfAdrh*, *CfDd*, *CfAct,* and *CfTub*) which were selected from *C. fructicola*-*Ca. oleifera* transcriptome data and evaluated and sequenced using geNorm, NormFinder, and BestKeeper algorithms. The results showed that *CfRrp* had better stability in *C. fructicola*, both during the growth of pure pathogenic fungi and during the invasion of different oil-tea leaves. After normalization with *CfRrp*, the differentially expressed target genes were similar to the transcriptome. Our work provides suitable reference genes for future studies to quantify target gene expression levels in *C. fructicola.*

## Introduction

*Colletotrichum* consists of a highly diverse group of pathogens that infect a wide variety of plants (Silva et al., 2017). According to statistics, under the influence of *Colletotrichum* spp., the quality of fruits exported from tropical, subtropical, and Mediterranean regions has declined significantly, causing economic losses of more than 80% (Joshi, 2018). The 2016 Global Plant Status Assessment Report lists *Colletotrichum* among the top 10 pathogenic fungi affecting plants globally (Royal Botanic Gardens, 2016). *Colletotrichum* has a highly diverse lifestyle and a wide variety of hosts, and studying the pathogenesis of *Colletotrichum* provides a reference for analyzing the interaction patterns between various plants and pathogenic(Silva et al., 2017). *C. fructicola* is the dominant pathogenic fungus that causes oil-tea anthracnose (Chen et al., 2021a; Tan et al., 2021), which causes flower drops, fruit, and leaf drop. It can lead to 20%–40% fruit drop and up to 40% loss of oil-tea seeds and also lead to the death of branches and even plants, causing significant economic losses and seriously hampering the oil-tea industry (Lee and Yen, 2006; Jin et al., 2009). *Camellia oleifera* Abel (oil-tea trees) is a unique woody oil plant in China, and its planting area is increasing yearly. The oil extracted from seed has high nutritional value and healthcare functions (Hong et al., 2019; Du et al., 2020).

According to the study of different functional genes, exploring a pathogen’s gene expression pattern (expression profile) at different stages is necessary. Due to its efficiency and sensitivity, Real-time quantitative PCR (RT-qPCR) is considered the most reliable technique for simultaneous measurement of relative levels of gene transcripts in a wide variety of samples (Galli et al., 2015; Machado et al., 2015). RT-qPCR has been used for the detection of pathogens such as bacteria, viruses, and fungi and for gene expression analysis in plant tissues and soil (Lopez et al., 2003; Schneider et al., 2012). However, RT-qPCR techniques must rely on data normalization using reference genes, and inappropriate reference genes may lead to skewed results (Hao et al., 2014; Pombo et al., 2019). Studies have shown that the stability of reference gene expression varies from species to species and may vary according to tissue type, developmental stage, and experimental conditions (Gutierrez et al., 2008; Guenin et al., 2009). For example, *Colletotrichum camelliae* has slightly different optimal reference genes during spore germination and mycelial growth and interaction with host plants (He et al., 2019). *C. fructicola*, as the dominant pathogenic fungus of oil-tea anthracnose, is widely distributed in oil-tea-producing areas. While there are wide varieties of oil-tea, the gene expression of *C. fructicola* under different conditions shows apparent differences. Therefore, using stably expressed reference genes is essential to provide a basis for mining broad-spectrum disease resistance genes in oil-tea.

In previous studies, *Colletotrichum* spp. mostly used housekeeping genes such as *Act*, *Tub*, and *GAPDH* as reference genes (Narusaka et al., 2009; Ben-Daniel et al., 2012; Kim et al., 2014). However, existing studies have shown that gene expression such as *Act* is unstable under certain conditions and cannot be used as reference genes for data normalization analysis (He et al., 2019). The discovery of this phenomenon has led more and more researchers to choose reference genes according to the actual situation of their research.

In this study, we evaluated the stability of eight candidate genes to identify the reference genes most suitable for *C. fructicola* to be used in conidia, mycelial growth, and infection of different oil-tea leaves. To evaluate the efficacy of selected reference genes, we used multiple sets of transcriptome data (mycelium, invasive oil-tea leaves 24, 48, and 72 h) to screen for candidate reference genes with minimal change in expression. The expression of the known effector CfEP92 in *C. fructicola* was normalized to the conidial period, the mycelial growth period and the process of infesting different oil-tea leaves using eight candidate reference genes and compared with the transcriptomic data to screen the suitable reference genes. To our knowledge, this is the first analysis of *C. fructicola* reference gene expression stability. Our work provides a theoretical reference for future researchers to analyze the gene function of *Colletotrichum* spp., and provides a basis for us to explore the key pathogenic genes of *C. fructicola*, explore its pathogenic mechanism, and study the interaction mechanism of *C. fructicola* and *Ca. oleifera* in the future.

## Materials and methods

### Plant and pathogen materials

The biennial seedlings of “Huajin” and “Huasuo” *Ca. oleifera* varieties (HJ and HS after the article) were selected as the test materials, respectively, and the two oil-tea leaves showed different resistance to *C. fructicola* (Supplementary Figure S1). Before inoculation, the two oil-tea seedlings were cultured indoors in an artificial climate of 24°C, 16 h light/8 h darkness, and 60%–80% relative humidity. *C. fructicola* is provided by the “Key Laboratory of the State Forestry Administration of Plantation Pest Control in the South.”

### Sample preparation

*Colletotrichum fructicola* stored in −80°C glycerin was inoculated on potato dextrose agar (PDA) medium and inverted at 28°C for 4 days (d). Five pieces of PDA with hyphae on the surface were cut and inoculated into a conical flask containing 200 ml potato dextrose broth (PDB) medium at 28°C and 180 rpm shaker cultures 5–6 days. Conidia were collected using 9 cm medium speed qualitative filter paper filtered into 50 ml sterile centrifuge tubes at 6,000 rpm/min centrifugation of 10 min. The concentration of ddH_2_O diluted spores to 1 × 10^6^ spores·ml^−1^ for inoculation of oil-tea leaves (Chen et al., 2021b), remaining conidia soaked up the water with sterile filter paper, liquid nitrogen refrigerated to −80°C for reserve.

Mycelium samples (JS after the article) culture in PDB medium shake flasks for 3 days and 6 days mycelium, and liquid nitrogen was refrigerated at −80°C for backup after moisture was soaked up in sterile filter paper.

Randomly pick HJ’s and HS’s several tender leaves, disinfect 10–15 s with 75% alcohol, rinse them twice in sterile water, then drain the surface with a sterile filter paper and place them in a sterile petri dish. Aseptic cotton absorbs the right amount of ddH_2_O and places it in a circle around the outside of the sterile dish to ensure ambient humidity. We used a sterile disposable syringe needle to puncture the surface of the above oil-tea leaves, and 10 μl diluted spore droplets were extracted and cultured in the middle of the hole at 28°C, with the control group receiving only sterile water. The leaf tissue with the disease spots was cut at 24, 48, and 72 h of *C. fructicola* infested oil-tea leaves and stored at −80°C under liquid nitrogen freezing. All treatments repeat three times.

### Total RNA extraction and reverse transcription

The leaves (HS-24, 48, and 72 h, HJ-24, 48, and 72 h), mycelium (JS-3 days, 6 days), and conidia were taken into a mortar, frozen in liquid nitrogen and well ground, and 10–20 μg of different sample powder were weighed and added to 1.5 ml sterile enzyme-free centrifuge tube, and different samples of RNA were extracted using RNAprep Pure Plant Plus Kit (Polysaccharides and Polyphenolics-rich; TianGen, Beijing, China). Extracted RNA was determined by Eppendorf Biophotometer D30 (Eppendorf, Hamburg, Germany) for purity and concentration. Sample RNAs were required with A260/A280 ratios of 1.9 to 2.1 and A260/A230 ratios of 19 to 2.0, indicating high purity and no protein contamination of samples. RNA integrity examines by 1% agarose gel electrophoresis. Approximately 100 ng of total RNAaspirate for cDNA was synthesized using the HiScript III 1st Strand cDNA Synthesis Kit (+gDNA wiper) kit (Vazyme, Nanjing, China).

### Selection candidate reference genes

Eight genes with moderate expression and little change over time were selected as candidate reference genes from *C. fructicola*-infected *Ca. oleifera* transcriptome data (Supplementary Table S1). The original RNA-seq readings were submitted to the NCBI under the BioProject PRJNA848256. To test the specificity of the selected genes, we performed general PCR using healthy HJ and HS leaf cDNA as templates for specific amplification. The PCR program consisted of a preliminary step of 2 min at 98°C followed by 35 cycles at 98°C for 10 s and 60°C for 10 s and 72°C for 10 s.

### Primer design

Complete information on BLAST candidate reference genes in NCBI. RT-qPCR primers design using Primer5 software.^1^ The length of the primer is 20 ~ 22 bp, the GC content is 45% ~ 55%, the melting temperature is 55°C ~ 60°C, the amplified fragment length is 100 ~ 250 bp, and it spans at least one intron (Table 1).

**Table 1:** Primer basic information of the candidate reference genes.

Reference genes	Gene ID	Function description	Forward/reverse primer sequence (5′-3′)	Amplicon size (bp)	Number of introns across
*CfTub*	CGMCC3_g2839	Tubulin beta-2 chain	GCCAGTGCGGTAACCAGATTG AGCTTCGTTGAAGTAGACGCTCATG	134	4
*CfActin*	CGMCC3_g6665	Actin	AGGTTGCTGCCCTCGTTATC CGCTTCGACTGTGCCTCATC	175	3
*CfDd*	CGMCC3_g11349	Dihydrolipoyl dehydrogenase	GTATCAACTTCAAGACCAGCACCAA GGCGACAAGGACAACTTCAGC	128	1
*CfCk*	CGMCC3_g11033	Casein kinase I 1	GCGATGCTCCTCAACTGCGAG GGCGACCATAACGACGGTCTT	197	1
*CfUce*	CGMCC3_g13714	Ubiquitin-conjugating enzyme	ATTAACAAGGAGCTCACTGACCTCG AGTCGGTAGGGAAGTGGATGGC	163	3
*CfRrp*	CGMCC3_g10154	Ras-related protein	GAGCGATTTGCGACACCTGC TTGCTGAGTGCGATGCTGGG	228	2
*CfAdRh*	CGMCC3_g2047	ATP-dependent RNA helicase	AGAGTCGGTCGTGCTGGTCGTT ATGTAGGTGCTGGCGTCAATG	155	1
*CfRpp*	CGMCC3_g12203	Ribose-phosphate pyrophosphokinase 2	AACTACTCCAACCAAGAAACCAGCG AGCGTAAGGAAAGCAAGGAATGAC	171	2

### Quantitative real-time PCR

Set 5 gradients 10 times diluted conidia cDNA to plot standard curves to calculate gene amplification efficiency (E) and correlation coefficient (R^2^).


$$E=\left(10^{\left(-\frac{1}{slope}\right)}-1\right)\times 100\%$$

RT-qPCR reactions were performed in 8 rows, each with a total volume of 15 μl:7.5 μl 2 × ChamQ Universal SYBR qPCR Master Mix (Vazyme, Nanjing, China), 0.6 μl forward primer(100 mol/l), 0.6 μl reverse primer (100 mol/l), 1 μl cDNA(50 ng), and 5.3 μl ddH_2_O. RT-qPCR analysis was performed on ABI Quant Studio Q3 (Thermo Fisher, Massachusetts, United States): 95°C Pre-denaturation for 30 s, followed by 40 cycles at 95°C for 10 s, 60°C for 30 s, and finally the fuse curve was detected. Each reaction consisted of three technical repeats and two NTCs (nuclease-free water-replacement cDNA). At the end of the RT-qPCR run, the products for primer specificity using 1% agarose gel electrophoresis.

### Statistical data analysis

The entire experiment process is conducted by Minimum Information for Publication of Quantitative Real-Time PCR Experiments (MIQE) requirements (Bustin et al., 2009).

The expression stability of candidate genes was assessed by quantitative cycle (Cq) value using three statistical algorithms, BestKeeper (Pfaffl et al., 2004), NormFinder (Andersen et al., 2004), and geNorm (Vandesompele et al., 2002).

*CfEp92* is known to induce expression as an effector of *C. fructicola* when infecting plant hosts (Shang et al., 2020), where the suitability of candidate reference genes is assessed and compared, with results calculated using the 2^−ΔΔCT^ method (Livak and Schmittgen, 2001).

Statistical analysis was performed using SPSS 25 software program. Notable difference analysis was performed by ANOVA and LSD test. Statistical significance is considered at ^*^p < 0.05 and ^**^p < 0.01. All experiments consisted of three technical repetitions and three biological replicates.

## Results

### Primer specificity and efficiency detection

In PCR assays modeled on cDNA from healthy HJ and HS leaves, none of the primers of candidate reference genes were amplified. In PCR assays using hyphal cDNA as a template, the primers of all candidate reference genes were amplified to obtain the target band.

**Figure 1:**
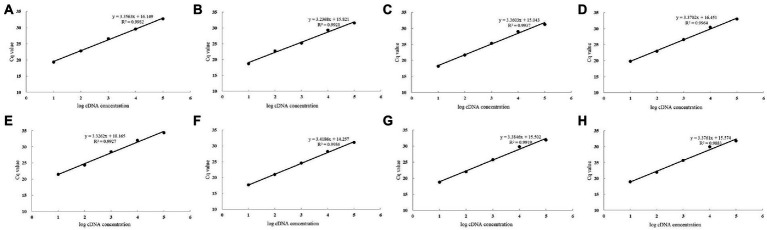
Standard curve of *Colletotrichum fructicola* RT-qPCR quantitative primers. The primer efficiency for the RT-qPCR quantification of the gene was determined using a serial dilution of cDNA templates from conidia. The respective correlation coefficients (R^2^) are indicated. **(A)** *CfTub*; **(B)** *CfAct*; **(C)** *CfDd*; **(D)** *CfCk*; **(E)** *CfRpp*; **(F)** *CfUce*; **(G)** *CfRrp*; and **(H)** *CfAdrh.*

All the primers were tested by RT-qPCR and the product was validated by 1% agarose gel electrophoresis as a single amplified product of desired size (Supplementary Figure S2), indicating that the cDNA template used was free of gDNA contamination. The results of the melting curve were all single peaks with no amplification of NTC response, indicating that no other secondary structures were generated (Supplementary Figure S3). Draw a standard curve (Figure 1) for all primers R^2^ > 0.98 with an amplification efficiency (E) value between 90% and 110% (Table 2).

**Table 2:** The primer amplification efficiency and correlation coefficients of candidate reference genes.

Reference genes	RT-qPCR efficiency (%)	R^2^	TM (°C)
*CfTub*	98.6	0.998	86.283
*CfAct*	103.7	0.992	88.065
*CfDd*	98.4	0.994	86.195
*CfCk*	98.0	0.996	84.698
*CfUce*	96.1	0.999	88.382
*CfRrp*	97.4	0.991	87.861
*CfAdRh*	97.8	0.988	85.614
*CfRpp*	99.8	0.993	88.245

### Expression levels of candidate reference genes

The quantitative cycle (Cq) value is the number of PCR cycles at the intersection of the sample response curve and the threshold line, which indicates how many cycles it takes to detect the true signal of the target gene from the sample, and the Cq value is inversely proportional to the number of target genes in the sample. Cq value in RT-qPCR can be used to compare gene expression levels, and the change in Cq value directly reflects gene expression stability. The qualified reference gene Cq value should keep relatively low in different samples to facilitate normalization of the target gene. Because of the low fungal content in the oil-tea leaves, fewer fungal active RNA can be extracted, resulting in significant differences in the Cq values detected in pure fungal samples compared to infected leaves. Therefore, analysis of candidate reference gene stability requires co-analysis in clusters, including pathogen sample (conidia, JS-3 days, JS-6 days) and infected leaf sample group (HS-24, 48, and 72 h, HJ 24, 48, and 72 h).

**Figure 2:**
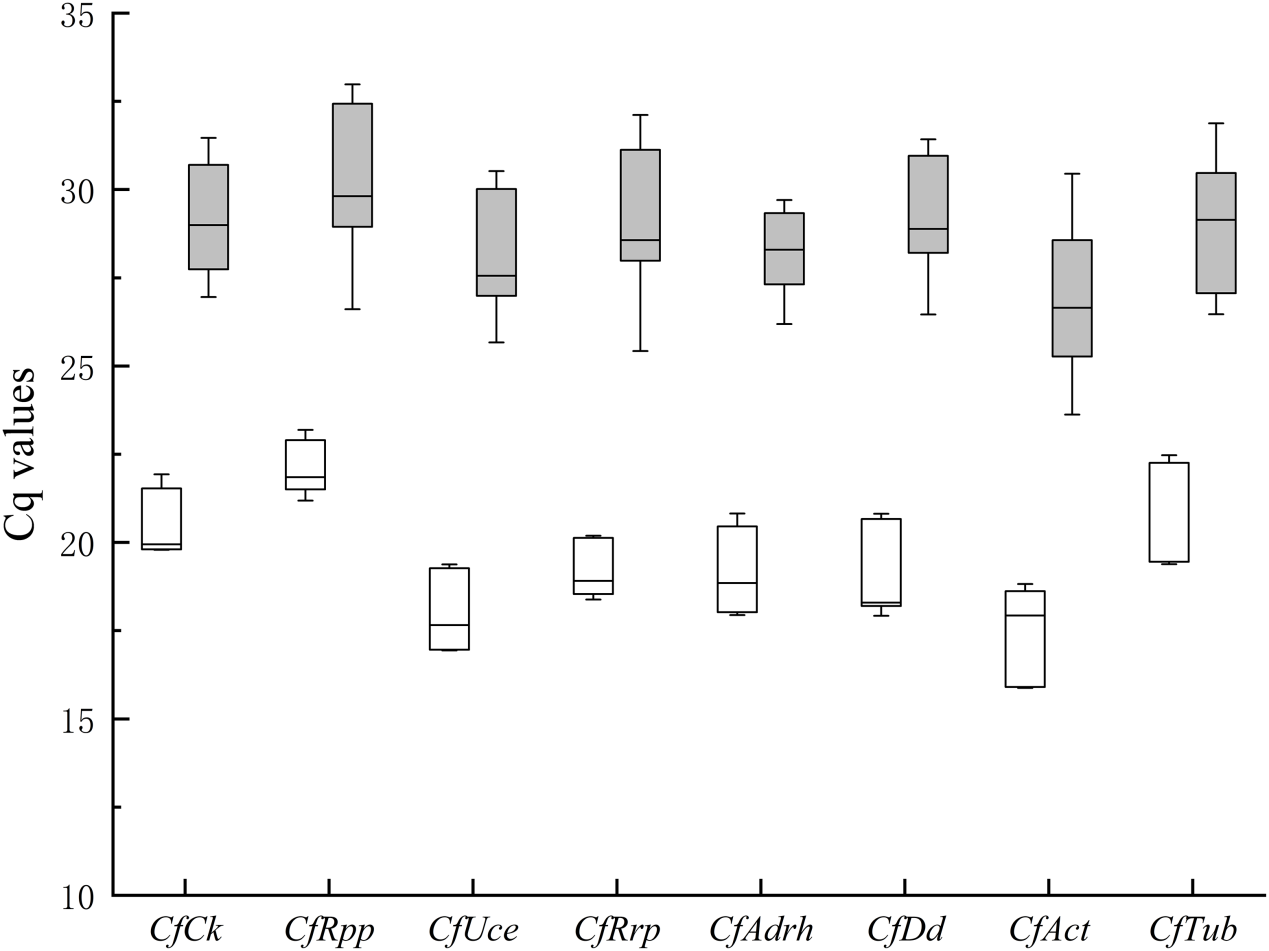
Expression levels of candidate housekeeping genes in pathogen and infected leaves. Boxes represent lower and upper quartiles of cycle thresholds range with medians indicated, and whisker caps represent maximum and minimum values. Hatched boxes correspond to pathogen samples (conidia, JS-3 days, 6 days) and white boxes to infected leaves samples (HS-24, 48, and 72 h, HJ-24, 48, and 72 h).

The results showed that *CfAct* expression was highest and *CfRpp* expression was lowest under all conditions. The expression of *CfRrp* and *CfRpp* in the pathogen sample group was the smallest, while the expression of *CfAct* was the largest. The expression of *CfAdrh* in the infected leaf sample groups was minimal, while the expression of *CfRpp* was the most variable (Figure 2). Observing the changes in Cq values, all gene expression differences in the infected leaf sample groups showed different degrees of increase, which predicted that certain genes were induced to be transcribed during the process of infesting oil-tea leaves, which would cause great errors during the normalized data processing if the reference genes were not carefully selected.

### Statistical analysis of RT-qPCR data by geNorm

To assess the stability of reference genes under different conditions, we subgroup eight candidate reference genes (*CfCk, CfRpp, CfUce, CfRrp, CfAdrh, CfDd, CfAct,* and *CfTub*), including pure pathogenic samples (conidia, JS-3 days, JS-6 days), HS leaf spot (HS-24, 48, and 72 h) and HJ leaf spot (HJ-24, 48, and 72 h).

**Figure 3:**
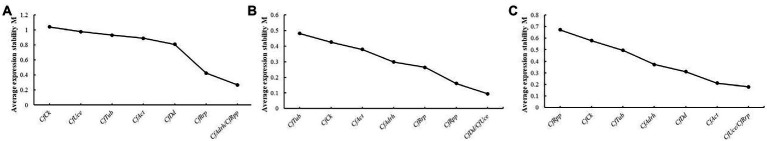
geNorm analysis of expression stability of eight candidate reference genes in different groups. **(A)** Mycelial growth; **(B)** HJ leaf spot group; and **(C)** HS leaf spot group. M values represent the gene expression stability index. Higher M values indicate that the gene is more unstable and vice versa.

The top 3 candidate reference genes were extracted from each group (the same M-value was considered equivalent rank), and the pure pathogen group: *CfRpp*, *CfAdrh*, *CfRrp*, and *CfDd* were the most stable; *CfDd*, *CfUce*, *CfRpp,* and *CfRrp* were the most stable in the HJ leaf spot group; *CfUce*, *CfRrp*, *CfAct,* and *CfDd* were the most stable in the HS leaf spot group (Figure 3). *CfRrp* and *CfDd* were detected in the stability evaluation of all three sets of data, which may indicate that both genes were stable under each condition.

### Statistical analysis of RT-qPCR data with NormFinder

**Figure 4:**
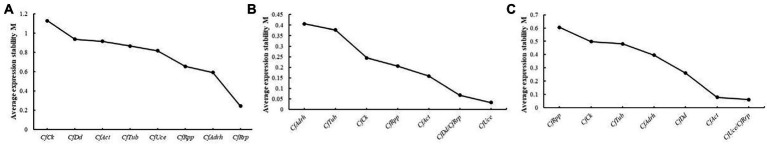
NormFinder analysis of expression stability of eight candidate reference genes in different groups. **(A)** Mycelial growth; **(B)** HJ leaf spot group; and **(C)** HS leaf spot group. M values represent the gene expression stability index. Higher M values indicate that the gene is more unstable and vice versa.

According to the NormFinder analysis, the top 3 candidate reference genes for stability were extracted from each group, with pure pathogen groups: *CfRrp*, *CfAdrh*, and *CfRpp* being the most stable. HJ leaf spot group: *CfUce*, *CfDd,* and *CfRrp* were the most stable; HS leaf spot group: *CfUce*, *CfRrp*, and *CfAct* were the most stable (Figure 4). Taken together, *CfRrp* was relatively stable under all conditions, consistent with the results of geNorm’s analysis, but *CfDd* did not enter the ranks of stable genes.

### Statistical analysis of RT-qPCR data with BestKeeper

**Figure 5:**
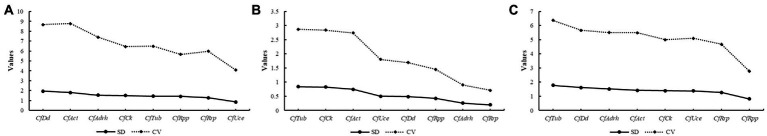
BestKeeper analysis of expression stability of eight candidate reference genes in different groups. **(A)** Mycelial growth; **(B)** HJ leaf spot group; and **(C)** HS leaf spot group. Standard deviation (SD) and coefficient of variation (CV) of Cq values for each gene were calculated. Stable reference genes have relatively low variability and standard deviation (CV ± SD).

Analysis of eight candidate reference genes revealed reference genes stability sequencing in the pure pathogen group: *CfUce > CfRrp > CfRpp > CfTub > CfCk > CfAdrh > CfAct > CfDd*; Reference genes stability sequencing in the HJ leaf spot group: *CfRrp > CfAdrh > CfRpp > CfDd > CfUce > CfAct > CfCk > CfTub*; Reference genes stability sequencing in the HS leaf spot group: *CfRpp > CfRrp > CfUce > CfCk > CfAct > CfAdrh > CfDd > CfTub* (Figure 5). Taken together, *CfRrp* was the most stable of the three groups.

### Evaluation of reference genes

To evaluate and compare the stability of different reference genes, we targeted their expression with a known effector *CfEP92*, as a target gene to detect its expression, and transcriptome data showed that the gene was induced expression at *C. fructicola* invasion of oil-tea leaves at 72 h, but less at other stages.

**Figure 6:**
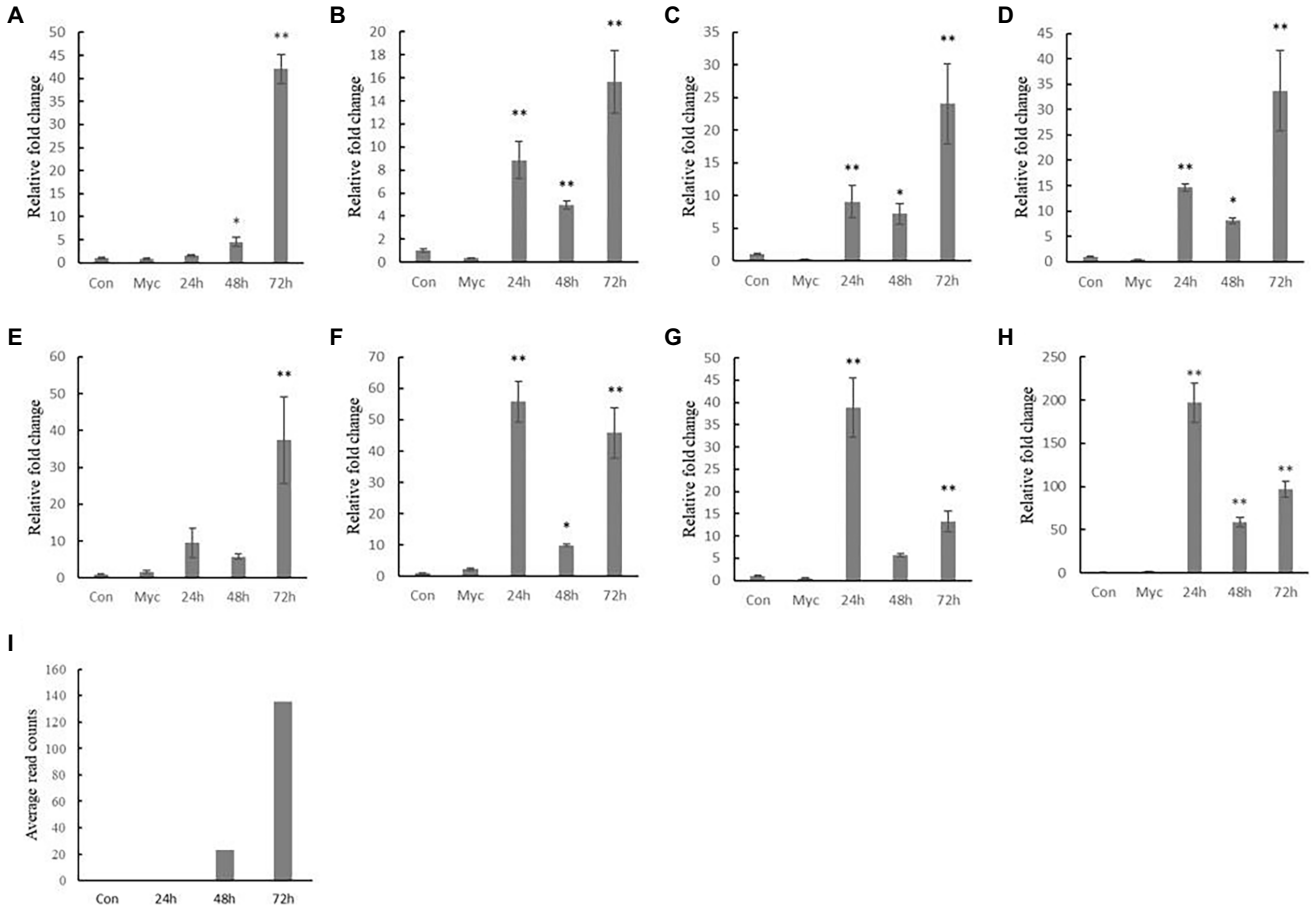
Expression profile of *CfEP92* in *Colletotrichum fructicola* conidia and hyphal growth and during 24, 48, and 72 h infection of tea-oil leaves. **(A)** *CfRrp*; **(B)** *CfAct*; **(C)** *CfTub*; **(D)** *CfUce*; **(E)** *CfRpp*; **(F)** *CfCk*; **(G)** *CfDd*; **(H)** *CfAdrh*; and **(I)** *CfEP92.* Con means Conidia; Myc means Mycelium. The significance analysis was compared with the conidial stage. The data show the mean expression ± standard deviation calculated from three biological replicates. ^∗^*p* < 0.05; ^∗∗^*p* < 0.01.

When *CfRrp* was used as a reference gene, *CfEP92* expression increased at 72 h in oil-tea leaves, with minimal expression in conidia and mycelium states. There were also significant differences at 24 and 48 h stages, with an overall trend of increasing expression, similar to transcriptome results (Figure 6). Targeted genes were even more expressed at 24 than 72 h using unstable reference genes such as *CfAdrh*, *CfDd,* and *CfCk*. When *CfAct*, *CfUce,* and *CfTub* were used as reference genes, the expression of target genes increased at 24 h. When *CfRpp* was used as reference gene, although the expression of target genes did not increase significantly at 24 h, there was no significant difference with 48 h, which was not consistent with transcriptome results.

## Discussion

As a dominant fungus strain on oil-tea leaves in China, *Colletotrichum fructicola* has severely impacted the *Ca. oleifera* industry. Studying and uncovering the function of vital pathogenic genes and differences in gene expression under different conditions is now vital to understanding the pathogenesis of pathogenic fungi (Skinner et al., 2001; Gao et al., 2017; Li, 2018). RT-qPCR is an essential technique for studying gene expression due to its sensitivity, accuracy, and repeatability, and the reliability of its results depends on the selection of appropriate reference genes (He et al., 2019; Song et al., 2019). Normalization of *C. fructicola* target genes expression using unevaluated reference genes may result in errors under specific experimental conditions. Therefore, we selected and evaluated the stability of *CfRrp*, *CfUce*, *CfRpp*, *CfTub*, *CfCk*, *CfAdrh*, *CfAct,* and *CfDd* genes during *C. fructicola* conidia, mycelium, and oil-tea leaves invasion provided a theoretical basis for *C. fructicola* pathogenic studies.

Reference gene needs to be expressed at a high level under different conditions, and in the RT-qPCR test, they are expressed at a Cq value. The eight candidate reference genes selected in this study maintained a high Cq value in all samples, and the Cq values remained below 35 even after 10,000-fold dilution. *CfAct* and *CfTub* are common housekeeping genes generally considered to have high expression in different tissues (Bustin et al., 2009). While the remaining 6 candidate reference genes were expressed nearly as much in the same tissue sample as both housekeeping genes, suggesting the potential of the eight candidate genes selected in this study as reference genes. All eight candidate reference genes had R^2^ values greater than 98%, and all candidate reference genes had sound linear amplification, while the amplification efficiency met the basic requirements of 90% ~ 110% (Bustin et al., 2009).

Since we screened reference genes that were stably expressed in *C. fructicola* invasive *Ca. oleifera* assays. In addition to conidia and pure mycelium, we also selected tissue samples from different oil-tea leaves. Both types of *Ca. oleifera* have different resistance to *C. fructicola.* Mycelium has slightly different growth and derivation rates in oil-tea leaf tissues, which may lead to changes in *C. fructicola* gene expression. Under this combination, the reference genes with better stability can be selected better. *CfUce* showed high stability in HJ leaf spot group and HS leaf spot group conditions but was less stable in pure mycelia, which is likely to be a functional gene that protects *C. fructicola* from persistent infection of oil-tea leaves. Retrospective *CfRrp* is stable in pure mycelium and also in HS leaf spot group and HJ leaf spot group.

In evaluating reference genes, the results of normalization of target genes by *CfRrp* are consistent with the transcriptome data. In previous studies of *C. fructicola* gene expression, housekeeping genes such as *Act* and *Tub* are often used to normalize (Liang et al., 2019; Song et al., 2019; Liu et al., 2021). *CfAct* and *CfTub* were not stable under the conditions set in this study, and the results were slightly different from transcriptome data after normalizing the target genes. Similarly, when other researchers evaluated reference genes, they found that management genes such as *Act* and *Tub* were unsuitable for all situations (Hao et al., 2014; He et al., 2019; Song et al., 2019). In conclusion, commonly used reference genes must be reconfirmed according to the specific experimental conditions. The use of unstable reference genes resulted in inaccurate or no significant difference in transcript level, for example, no significant difference in expression between 24 h and 48 h after *CfRpp* normalized target genes. After *CfAdrh* normalized the target genes, the expression of 24 h was significantly higher than 72 h. These results were not consistent with transcriptome data. So, the use of reliable reference genes is a prerequisite for the accurate analysis of RT-qPCR data.

## Conclusion

This is the first report to evaluate reference genes suitable for RT-qPCR analysis in *C. fructicola*. We screened eight candidate reference genes using transcriptome data and assessed their stability for normalization of *C. fructicola* gene expression. Through three computer algorithms geNorm, BestKeeper, and NormFinder, the results show that *CfRrp* was the most stable reference gene for conidia, mycelium, and the interaction between pathogens and *Ca. oleifera*. Analysis of *CfRrp* expression confirmed the importance of the selection of appropriate reference genes for standardizing RT-qPCR data. The reference genes selected here provide essential options for *C. fructicola* target gene expression and function studies.

## Data availability statement

The original contributions presented in the study are included in the article/Supplementary material, further inquiries can be directed to the corresponding authors.

## Author contributions

JL designed the experiments. QT and YH performed the experiments. GZ and ZW analyzed the data. XingzC and XinggC wrote the manuscript. All authors contributed to the article and approved the submitted version.

## Funding

This research was funded by the National Natural Science Foundation of China (31971661); Natural Science Foundation of Hunan Province (2021JJ31145); Forestry Science and Technology Innovation Fund Project of Hunan Province (XLK202101-2); and *Camellia oleifera* Industrial Research Demonstration Project of Hunan Province (XiangCaiZiHuanZhi [2021] No. 9).

## Conflict of interest

The authors declare that the research was conducted in the absence of any commercial or financial relationships that could be construed as a potential conflict of interest.

## Publisher’s note

All claims expressed in this article are solely those of the authors and do not necessarily represent those of their affiliated organizations, or those of the publisher, the editors and the reviewers. Any product that may be evaluated in this article, or claim that may be made by its manufacturer, is not guaranteed or endorsed by the publisher.

## Supplementary material

The Supplementary Material for this article can be found online at: https://www.frontiersin.org/articles/10.3389/fmicb.2022.982748/full#supplementary-material

## Footnotes

^1^ http://www.premierbiosoft.com/primerdesign/
