# Supplementary material for: Selection of potential reference genes for RT-qPCR in the plant pathogenic fungus Colletotrichum fructicola
Source: Front Microbiol. 2022 Aug 8;13:982748. doi: 10.3389/fmicb.2022.982748 (PMC9393503; doi:10.3389/fmicb.2022.982748)
Supplement: Supplementary file 1 [file Data_Sheet_1.docx]

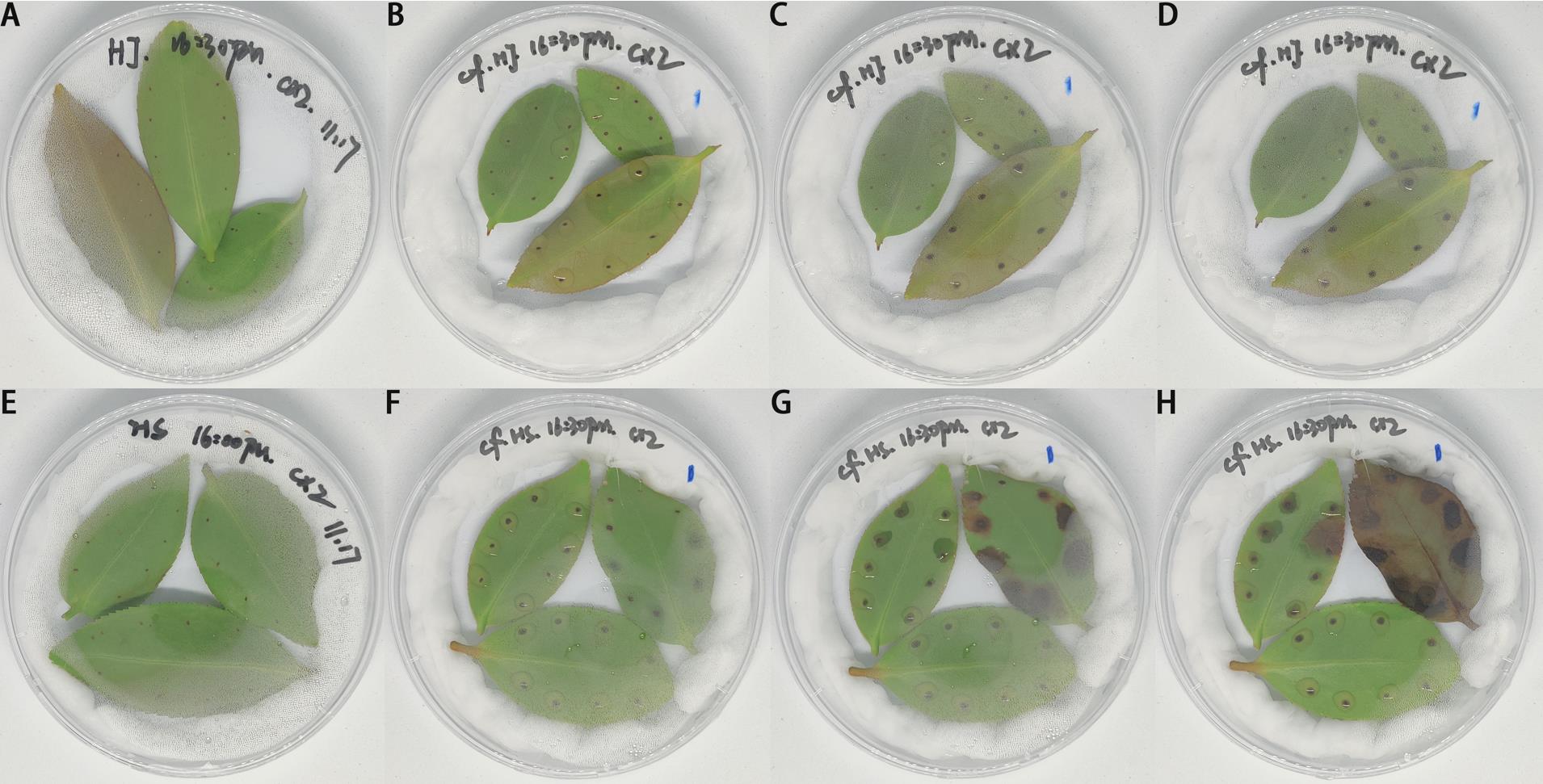
Supplementary Figure 1. Experiment of C. fructicola infecting different tea-oil leaves. A：“Huajin”blank control, distilled water replaced spore fluid；B-D：Spore fluid infected “Huajin”leaves for 24h, 48h and 72h；E：“Huasuo”blank control, distilled water replaced spore fluid；F-H: Spore fluid infected “Huasuo”leaves for 24h, 48h and 72h.


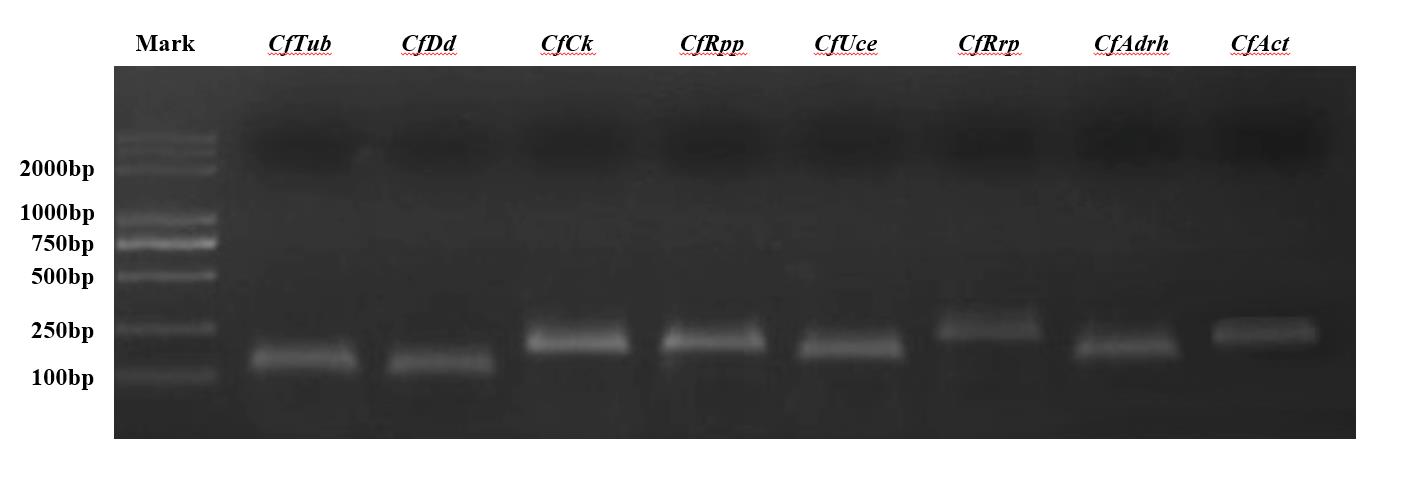
Supplementary Figure 2. Electrophoretic detection of RT-qPCR products of 8 candidate reference genes.


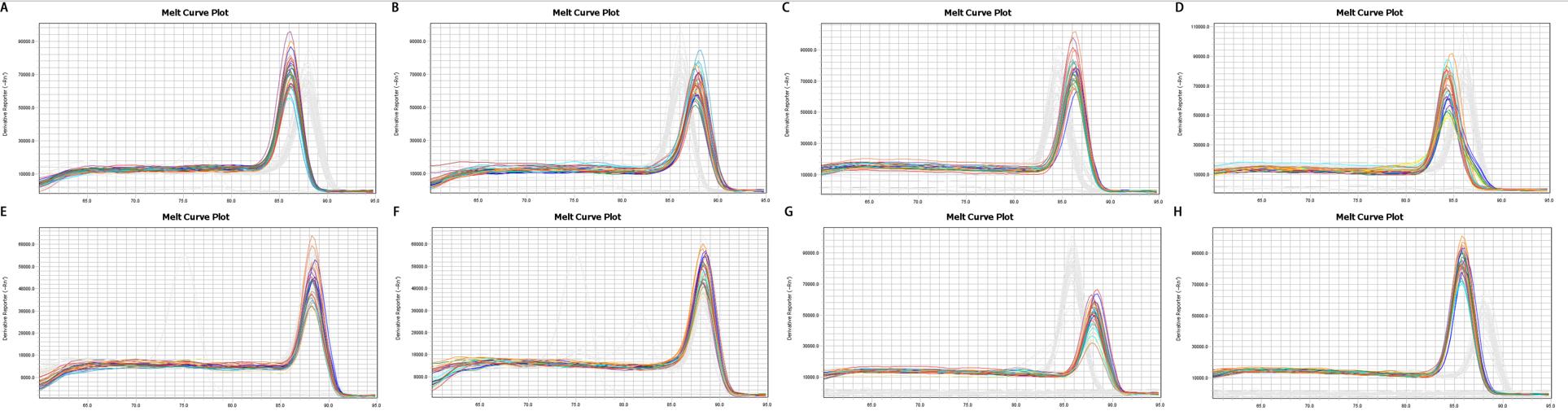
**Supplementary Figure 3.** Melting curve analysis of 8 candidate reference genes. All RT-qPCR products had a single melting curve indicating the breakdown of only one PCR product. **(A)***CfTub*;**(B)***CfAct*;**(C)***CfDd*;**(D)***CfCk*;**(E)***CfRpp*;**(F)***CfUce*;**(G)***CfRrp*;**(H)***CfAdrh*

**Supplementary Table S1.** The counts of candidate reference genes from *Colletotrichum fructicola* infected *Camellia oleifera* transcriptome data

| Gen | 24h-1 | 24-2 | 24h-3 | 48h-1 | 48h-2 | 48h-3 | 72h-1 | 72h-2 | 72h-3 | Con-1 | Con-2 | Con-3 |
| --- | --- | --- | --- | --- | --- | --- | --- | --- | --- | --- | --- | --- |
| *CfTub* | 193.91 | 260.98 | 241 | 407.03 | 502.48 | 377.76 | 319.78 | 493.29 | 241.04 | 323.61 | 371.67 | 294.95 |
| *CfAct* | 1315.2 | 921.41 | 1497.87 | 985.15 | 941.18 | 900.87 | 877.46 | 1189.31 | 976.36 | 890.94 | 1027.05 | 1051.35 |
| *CfDd* | 162.61 | 250.01 | 214.1 | 241.61 | 245.91 | 219.24 | 289.8 | 219.11 | 227.95 | 264.54 | 278.45 | 240.91 |
| *CfCk* | 201.48 | 135.11 | 199.09 | 340.16 | 281.92 | 207.42 | 190.15 | 141.96 | 271.07 | 444.67 | 355.28 | 305.83 |
| *CfUce* | 871.71 | 734.7 | 607.63 | 655.95 | 700.41 | 908.24 | 810.75 | 829.14 | 642.93 | 663.18 | 697.93 | 701.84 |
| *CfRrp* | 336.53 | 220.38 | 292.47 | 376.57 | 326.79 | 396.05 | 248.96 | 296.82 | 223.54 | 319.13 | 318.8 | 322.69 |
| *CfAdRh* | 216.02 | 197.17 | 266.94 | 231.18 | 178.17 | 202.92 | 246.79 | 171.21 | 155.52 | 218.75 | 245.55 | 218.34 |
| *CfRpp* | 176.42 | 213.68 | 165.76 | 337.26 | 355.04 | 342.84 | 220.94 | 322.73 | 327.97 | 140.92 | 113.43 | 138.27 |
| *CfEP92* | 0 | 0 | 0 | 70.07 | 0 | 0 | 31.6 | 72.19 | 302.44 | 0 | 0 | 0 |
